# Reinforcement of Alginate-Gelatin Hydrogels with Bioceramics for Biomedical Applications: A Comparative Study

**DOI:** 10.3390/gels7040184

**Published:** 2021-10-26

**Authors:** Alan Avila-Ramirez, Kevin Catzim-Ríos, Carlos Enrique Guerrero-Beltrán, Erick Ramírez-Cedillo, Wendy Ortega-Lara

**Affiliations:** 1 Tecnologico de Monterrey, Escuela de Ingeniería y Ciencias, Av. Eugenio Garza Sada 2501 Sur, Monterrey 64849, Mexico; alan.avilaramirez@kaust.edu.sa (A.A.-R.); catzimkevin@gmail.com (K.C.-R.); erickramce@tec.mx (E.R.-C.); 2 Division of Biological & Environmental Science & Engineering (BESE), King Abdullah University of Science and Technology (KAUST), Thuwal 23955-6900, Saudi Arabia; 3 Tecnologico de Monterrey, Escuela de Medicina y Ciencias de la Salud, Medicina Cardiovascular y Metabolómica, Monterrey 64710, Mexico; enriqueguerrero@tec.mx

**Keywords:** biomaterials, biopolymer, bioceramics, bone, cartilage, restoration

## Abstract

This study states the preparation of novel ink with potential use for bone and cartilage tissue restoration. 3Dprint manufacturing allows customizing prostheses and complex morphologies of any traumatism. The quest for bioinks that increase the restoration rate based on printable polymers is a need. This study is focused on main steps, the synthesis of two bioceramic materials as *WO*_3_ and *Na*_2_*Ti*_6_*O*_13_, its integration into a biopolymeric-base matrix of Alginate and Gelatin to support the particles in a complete scaffold to trigger the potential nucleation of crystals of calcium phosphates, and its comparative study with independent systems of formulations with bioceramic particles as *Al*_2_*O*_3_, *TiO*_2_, and *ZrO*_2_. FT-IR and SEM studies result in hydroxyapatite’s potential nucleation, which can generate bone or cartilage tissue regeneration systems with low or null cytotoxicity. These composites were tested by cell culture techniques to assess their biocompatibility. Moreover, the reinforcement was compared individually by mechanical tests with higher results on synthesized materials *Na*_2_*Ti*_6_*O*_13_ with 35 kPa and *WO*_3_ with 63 kPa. Finally, the integration of these composite materials formulated by Alginate/Gelatin and bioceramic has been characterized as functional for further manufacturing with the aid of novel biofabrication techniques such as 3D printing.

## 1. Introduction

Actual technologies allow intricate and customizable designs for implants. Biofabrication coming from state-of-the-art additive manufacturing techniques such as 3D printing are novel technologies that offer reproducibility, easy printer handling, evade conventional molds, avoid raw material waste, and can be operated remotely [1,2]. Hydrogels have demonstrated to be an excellent alternative for tissue replacement; therefore, several natural polymers such as collagen, chitosan, gelatin, silk are under development for many biomedical applications in the tissue engineering domain [3,4]. Additionally, its physicalchemical properties such as viscosity, pH, cross-linking, and concentration will allow these systems to be manufactured in 2D and 3D structures, especially in extrusion-based 3D printers, allowing them to pass the formulated inks through extrusion channels as needles or tips [5]. Respecting biocompatibility and interaction with the patient requires appropriate mechanical properties, accurate degradation time in the in vivo host’s function, and biomimetic ability depending on the specific cell population and tissue under study [6,7,8].

The benefits of these hydrogels-based inks are the possibility of generating complex structures that mimic specific parts of the human body according to patients’ particular needs. These materials allow the unique designs of complex systems and appropriate mechanical properties, measured by its elasticity module or intrinsic strength depending on the tissue that needs to be addressed [9,10,11]. The purpose of tissue regeneration is to produce specific biological substitutes for patients to counteract the limitations in treating damaged tissues, such as the scarcity of replacement, chronic rejection, cytotoxicity, or cellular non-proliferation [12]. The hydrogel interaction in the targeted tissue requires appropriate mechanical properties, accurate time of degradation, and biomimetic properties depending on the specific tissue in which it is working [13,14,15]. Alginates are naturally derived linear copolymers of 1,4-linked ß-D-mannuronic acid and a-L- guluronic acid residues that form crosslinked structures in the presence of divalent cations. Their hydrogels are soft, dissipative materials and are mainly studied for biomedical and pharmaceutical applications [16]. One of the blends that have caught the attention of studies in drug delivery and tissue engineering effects is alginate/gelatin to enhance the properties of the synthesized hydrogel. On the other hand, gelatin is used widely in biomedical applications; this is a thermoresponsive polymer that undergoes reversible sol-gel transition, which depends not only on temperature but also on the concentration of the gelatin solution [17]. In previous works, alginate/gelatin systems showed excellent performance during the extrusion process, presenting porous polymeric nets combined with bioactive fillers or bioceramics, which can stimulate apatite nucleation for tissue regeneration [17,18]. According to Urruelas et al., viscoelastic studies showed a higher storage modulus than the loss modulus at low shear rates in the Alginate-Gelatin system (3.5:0.8% *w*/*v*) with a 0.2 *w*/*v*% CaCl_2_—in this case, when G’ > G” avoids the collapse of the materials once printed. Furthermore, adding 1% nanoparticles does not seem to affect the solution behavior [19]. Therefore, bioceramics as Titanium hexatitanate (*Na*_2_*Ti*_6_*O*_13_), Zirconium oxide (*ZrO*_2_), Aluminum oxide (*Al*_2_*O*_3,_), and Titanium oxide (*TiO*_2_) and Tungsten oxide (*WO_3_*) particles can potentially promote the hydroxyapatite nucleation, and enhance its mechanical properties depending on the desired targeted tissue [17,20,21,22]. In this study, the stated *Al*_2_*O*_3_, *Na*_2_*Ti*_6_*O*_13_, *TiO*_2_, *ZrO*_2_ and *WO*_3_ bioceramics were embedded in an alginate-gelatin matrix and characterized physicochemically. Bioactivity evaluation is a crucial opportunity to scale the system to its implementation to have a specific alternative to the lack of tissue donors and at the same time counteract diseases of a bone nature such as osteoporosis [23]. The bioceramics particles are expected to reduce toxicity and optimize cellular proliferation in time and quantity by generating hydroxyapatite [24,25,26].

## 2. Methodology

The following materials and reactants were used to formulate the polymer base and the necessary particles for the reinforcement and subsequently realize the fundamental characterizations. Titanium (IV) propoxide (97%, Sigma-Aldrich, Saint Louis, MO, USA), tert-butanol (>99%, Sigma-Aldrich, Saint Louis, MO, USA), Gelatin (cold water fish skin, Sigma-Aldrich, Saint Louis, MO, USA), hydrochloric acid, (37%, Sigma-Aldrich, Saint Louis, MO, USA), sodium tungstate dihydrate (99%, Sigma-Aldrich, Saint Louis, MO, USA) titanium dioxide(20 nm, Sigma-Aldrich, Saint Louis, MO, USA), dialuminum trioxide (21 nm., Sigma-Aldrich, Saint Louis, MO, USA) and zirconium (IV) dioxide (Sigma-Aldrich, Saint Louis, MO, USA), sodium acetate, (99%, Sigma-Aldrich, Saint Louis, MO, USA), sodium nitrate (>99%, Sigma-Aldrich, Saint Louis, MO, USA), potassium chloride (99–105%, Vetec, Saint Louis, MO, USA) anhydrous sodium sulfate (>99%, Sigma-Aldrich, Saint Louis, MO, USA), trizma base (>99.9%, Sigma Aldrich, Saint Louis, MO, USA), dibasic potassium phosphate (>98%, Sigma-Aldrich), sodium bicarbonate (100188, CTR Scientific, Monterrey, Mx), magnesium chloride hexahydrate (99–102%, Sigma-Aldrich, CTR Scientific, Monterrey, Mexico), potassium chlorate (99–100.5%, Vetec, Saint Louis, MO, USA), sodium chloride (99%, Vetec, Saint Louis, MO, USA), magnesium chloride (99–102%, Monterrey Chemicals), phosphate buffered saline (Sigma-Aldrich, Saint Louis, MO, USA), deionized water, and milliQ water(Filters from FEMSA biotechnology center)

### 2.1. Particles Synthesis

This synthesis method with disodium hexatitanate uses tert-butanol (>99%, Aldrich), sodium acetate (99.99%, Aldrich), and the precursor of inorganic ion is titanium isopropoxide (IV) (97%, Aldrich). It is added until it dissolves in a 10:1 volume relation with the solvent and 2:1 with deionized water. After obtaining the homogenous and crystalline solution, it is added to the titanium precursor dropwise. It is necessary to declare that this step must be made in an environment far from the light because titanium isopropoxide (IV) is photosensitive and reacts with light interaction. A thermic treatment is applied in an oil bath for 24 °C at 70 °C with constant stirring. Once treated, the solvent is evaporated at a temperature of approximately 40 °C, the wet gel is dried at 80 °C by 48 h and proceeds to spray the sample in a crucible, taking portions of the sample and calcining at 200 °C, 400 °C, 600 °C, 800 °C, 1000 °C, and 1200 °C, to be previously characterized and used in future protocols, presented in Figure S1 (XRD), Figure S2 (TGA), and Figure S3 (FT-IR) [27,28].

Tungstate oxide was obtained by precipitation, followed by a thermal treatment in an oil bath. Every 5 g sodium tungstate dihydrate a WO₄*2(H₂O) (99%, Sigma-Aldrich) as a precursor of the tungsten ion, dissolving in 150 mL of water free of salts, either deionized or milliQ to form a transparent solution. Hydrochloric acid (37%) is added dropwise until it becomes crystalline yellow. The above solution is treated at a constant temperature of 95 °C for 24 h until a white-yellow precipitate of sodium tungsten oxide hydrates ((Na_0.17_WO_3.085_)(H₂O)_0.17_). The remaining powder WO_3_ powder was obtained by drying the precipitate at 110 °C and then calcined at 500 °C for five hours (the heating speed at approximately 5 °C min^−1^), with a speed of cooling speed of about 2 °C min^−1^) in a flask, characterization at Figure S4 (XRD), Figure S5 (TGA), and Figure S6 (FT-IR) [29].

### 2.2. Hydrogels Preparation

The preparation method integrates the biopolymers Alginate 3.5% and Gelatin 0.88% (*w*/*w*) into milliQ water, after stirring at 45 °C/2 h constantly. The pre-crosslinked solution (0.2% *w*/*v* of calcium chloride) is extruded into specific morphology and then laid down into a 6% *w*/*v* calcium chloride solution for hardening the material. However, ionic crosslinking acts immediately. It is recommended to leave the material in the solution for more extended periods, depending on the sample’s morphology and size, which could go from minutes to hours [19,30].

The Alginate/Gelatin polymer-based is stirred as a pre-crosslinked polymer at 45 °C/2 h; 1% of bioceramics was added to the alginate/gelatin and stirred for an hour, and the mix was ultrasonicated to obtain a homogeneous solution [31]. The composition of hydrogel-bioceramics systems is presented in Table 1.

### 2.3. Instrumentation for Chemical and Mechanical Characterization

X-ray diffraction powder. XRD analyses of biocomposites were assessed in a PANalytical Empyrean diffractometer (PANalytical, Almelo, The Netherlands) with a 2θ between 10–80° with CuK_α_ radiation with a 45 kVA and a current of 40 mA. Mechanical Properties. The Instron 3360 universal machine analyzes cross-linked reinforced hydrogels at 1 mm/min to obtain the average modulus of elasticity measurement. Fourier-transform infrared spectroscopy. Perkin Elmer Spectrum 400 spectroscope equipment was used for FT-IR (Waltham, MA, USA) recorded in the wavelength range between 400 cm^−1^ to 4000 cm^−1^ at room conditions. Thermogravimetric analysis. TGA was performed in a Perkin–Elmer Pyris 8000 equipment (Monterrey, México) with a heating rate of 10 C/min from 50–700 °C. Microscopy analysis was observed in an EVO MA25 Zeiss Scanning electron microscope (Zeiss, Jena, Germany). The conditions were an accelerating voltage of 20 kV, and high vacuum samples were previously coated with carbon. Dynamic light scattering (Malvern Panalytical–Zetasizer), sometimes called photon correlation spectroscopy or near-elastic light scattering, is a technique for measuring particle size, measuring Brownian motion; this phenomenon corresponds to the random movement of the particles due to the bombardment of the molecules in the solvent that surrounds them.

### 2.4. 3D Printing

The additive manufacturing technique of 3D extrusion-based printing was used for the composites of Alginate/Gelatin hydrogels with nanoparticles with the assistance of custom-designed bioprinter with double extrusion processes (1) thermoplastic extrusion and (2) piston mechanical motor extrusion (3D Biofactory, 3D FACTORY MX, México). The mechanical piston motor was used for the process due to the materials’ shear-thinning properties [32]. A cylinder of 20 mm in diameter and 3 mm in height was designed in Solidworks 2021 and introduced to Cura (Ultimaker, The Netherlands) to generate the g-code. A syringe of 10 mL was mounted in the extrusion system with a cylindrical needle of 0.43 mm. Printing parameters were a layer height of 0.1 mm, a dispensing speed of 0.4 mm/s, an infill line distance of 5 mm; extrusion flow was varied from 40%, with no addition of heat during the extrusion process [33,34,35].

### 2.5. Cell Culture and Cell Viability Assay

Endothelial cells C166 line (ATCC^®^ CRL-2581TM) were cultured using DMEM (Dulbecco’s Modified Eagle’s Medium, Sigma, D7777, Saint Louis, MO, USA ) as culture medium, supplemented with 10% FBS (Fetal Bovine Serum, Biowest, S1650) and streptomycin (100mg/mL; GIBCO, 15140, Inc. Grand Island, NY, USA). Cells were incubated in 95% air-5% CO_2_ at 37 °C. Cell culture passages were performed when they reached 80% confluence. To determine the toxicity of the selected materials composite of Alg/Gel and bioceramic in endothelial cells, C166 cells were cultured at 1 × 10^3^ cell/mL in 96-well plates and incubated for 24 h with different experimental conditions. Control (incubated only with DMEM) and different concentrations of the selected composites or free Al_2_O_3_ were resuspended, sonicated, and incubated within the cells with DMEM. As an initial test, the toxicity of the different composites and particles were studied at concentrations of 100, 500, and 1000 μg/mL. Triplicates of each of these experiments were generated for statistical analysis of the results. To determine cell viability after the time of treatment with the experimental groups (after 24 h exposure with composite of Alg/Gel and bioceramic), the cells were incubated with Alamar blue (Sigma-Aldrich, Saint Louis, MO, USA) (1:10) for 3 h at 37 °C. This procedure measures the metabolic activity of cells through their ability to reduce resazurin (Alamar blue) to resorufin (its fluorescent form) by oxide reductases that are mainly found in the mitochondria of living cells, which can be quantified through spectrophotometry at wavelengths of 560 and 590 nm for excitation and emission, respectively. Cell viability assay: ANOVA data were analyzed, followed by Dunnett’s multiple comparisons tests using Graph Pad InStat (Graph Pad Software, San Diego, CA, USA). Data were expressed as means ± SD. A *p*-value of <0.05 was considered statistically significant.

## 3. Results and Discussion

**Figure 1:**
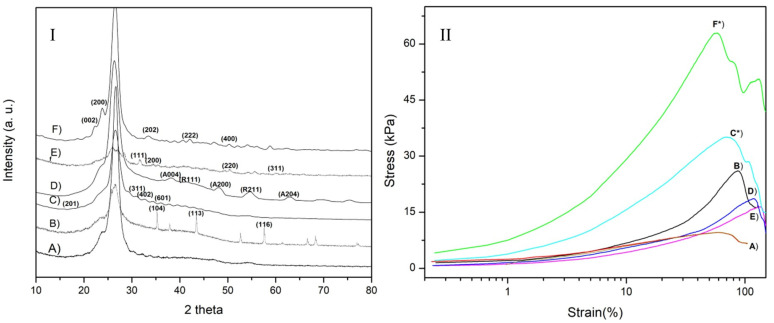
(**I**) XRD Alginate-gelatin hydrogels (A) AG1 (control); (B) AG2 (Al_2_O_3_); (C) AG3 (Na_2_Ti_6_O_13_); (D) AG4 (TiO_2_); (E) AG5 (ZrO_2_); and (F) AG6 (WO_3_); (**II**) Mechanical assays of alginate-gelatin samples (A) AG1 (control); (B) AG2 (Al_2_O_3_); (C) AG3* (Na_2_Ti_6_O_13_); (D) AG4 (TiO_2_); (E) AG5 (ZrO_2_); and (F) AG6* (WO_3_).

X-ray diffraction. XRD studies were assessed on samples previously dried. In Figure 1I, diffractograms of alginate/gelatin with 1% bioceramics added are observed (Figure 1 (IA–IF)). A semicrystalline curve is observed in 1IA, the mixture of porcine gelatin and sodium alginate presents typical noise in the curves, and prominent peaks are observed at 23 and 26° [36]. The Figure 1(IB) curve shows principal peaks due to the presence of crystalline corundum, also called sapphire M = 101.96 g/mol and ρ = 3.98 g/cm^3^ (JCPDS: 01-076-7774; 35.35, 43.46, 57.65°; Al_2_O_3_) [37]. The 1IC curve shows the highest representative intensities related to sodium hexatitanate, M = 301.58 g/mol, ρ = 2.484 g/cm3. overlapped with alginate and gelatin signals, (JCPDS: 01-081-9759; 17.37, 31.06, 32.62, 36.07°; Na_2_Ti_6_O_13_). Curve 1ID displays peaks of composite particles of titanium dioxide, anatase (JCPDS; 01-075-8897; 38.34, 48.23, 63.02°; anatase TiO_2_) and rutile (JCPDS; 01-079-6029; 54.67, 41.43°; rutile TiO_2_) M = 79.866 g/mol, ρ = 4.23 g/cm^3^. Curve 1IE shows that, besides the alginate-gelatin matrix, the highest intensities of zirconia are presented (JCPDS: 31.67, 34.41; 50.37° cubic ZrO_2_): M = 123.218 g/mol, ρ = 5.68 g/cm^3^ for some intensities for tetragonal zirconia. Finally, diffractogram 1IF of particles embedded in the alginate-gelatin matrix confirmed the presence of tungstate oxide particles (JCPDS: 00-024-9747; 23.98, 22.67, 33.46°; tetragonal WO_3_), M = 231.84 g/mol, ρ = 7.16 g/cm^3^. This study served to present an accurate definition of all the compounds of the different bioinks at crystalline point of view, in order to provide an accurate state from the bioceramic compounds used for our formulations.

Mechanical properties. Samples were rehydrated, and mechanical assessment is observed in Figure 1II. Control behavior (AG1) in (IIA), the lowest of the set. In general, samples reinforced with 1% of particles exhibit better performance than non-reinforced hydrogel. The samples IID and IIE present a good deformation and increase the strength to 16 and 18 kPa. The sample reinforced with alumina (IIB) shows a higher strength and similar performance than IIA without any reinforcement. A strengthening is observed of composites (IIC) and (IIF) (sodium hexatitanate and tungstate oxide), and 35 and 63 kPa results agreed with previous studies [19]. Therefore, our synthesized particles that have the highest molecular weights provide a higher mechanical strength, which could be applied for rigid-living systems such as cartilage, parts of bone tissues, or even from other rigid biological entities [38].

**Figure 2:**
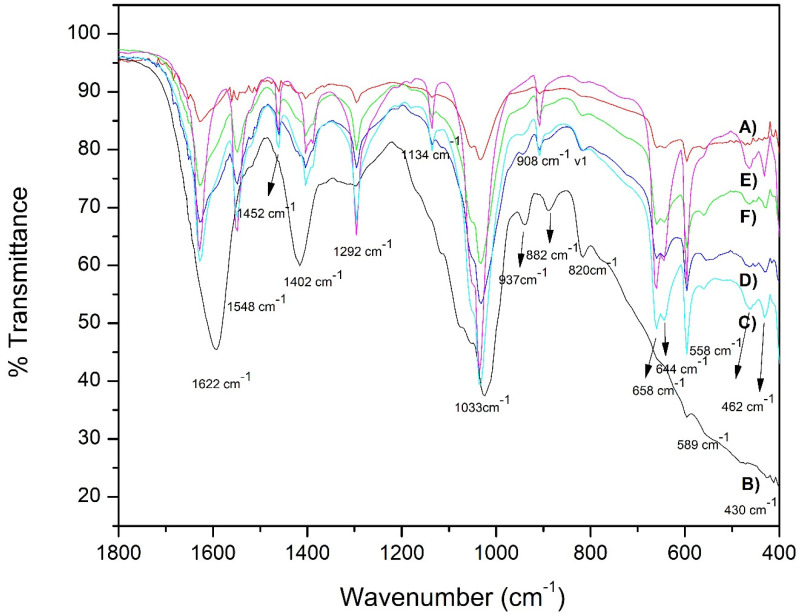
FT-IR of samples soaked for three weeks in simulated physiological fluids, (A) AG1 (control); (B) AG2 (Al_2_O_3_); (C) AG3 (Na_2_Ti_6_O_13_); (D) AG4 (TiO_2_); (E) AG5 (ZrO_2_); and (F) AG6 (WO_3_).

Infrared spectroscopy. In Figure 2, the apatite nucleation is observed in samples soaked for three weeks in simulated body fluids. All samples showed main characteristic bands, at low wavenumbers 430, 462 cm^−1^ [PO_4_^3−^] υ2 vibration. At 548, 644, and 658 cm^−1^ [PO_4_^3−^], υ4 vibration types occur and 937 cm^−1^ vibration type [PO_4_^3−^] υ1 [39]. A large band is observed in all samples at 1033 cm^−1^, a bending mode of PO_4_ [39,40]. At 882, a band related to HPO_4_^2−^ appears, usually characterizes Hap with calcium deficiency. 1452, 1530, and 1622 cm^−1^ are located bands due to CO_3_^2−^ in Hap type B [41]. Type B- apatite substitution is a replacement in phosphate groups that promotes bone regeneration and a higher solubility [42]. Infrared analysis as a semiquantitative technique reflected that some samples observed larger transmittance apatite bands as follows: AG2 > AG3 > AG4 > AG6 > AG5 > AG1. EDS analysis confirmed the presence of compounds rich in calcium and phosphorus in Table 2. However, Ca/P ratios are far from bone ratio at 21 days soaked in simulated body fluids (1.67). Therefore, the interaction of the formulations after three weeks in a simulated physiological fluid arrows and important insight of a potential nucleation of apatite-like crystals, which is crucial for bone-tissue engineering methodologies. On the other hand, FTIR studies were used to corroborate the correct synthesis of Na_2_Ti_6_O_13_ and WO_3_; therefore, different measurements were temperatures of calcination presented in the Supplementary Information (Figures S1–S6), which states that Na_2_Ti_6_O_13_ and WO_3_ must be calcined at 1200 °C and 500 °C, respectively.

**Figure 3:**
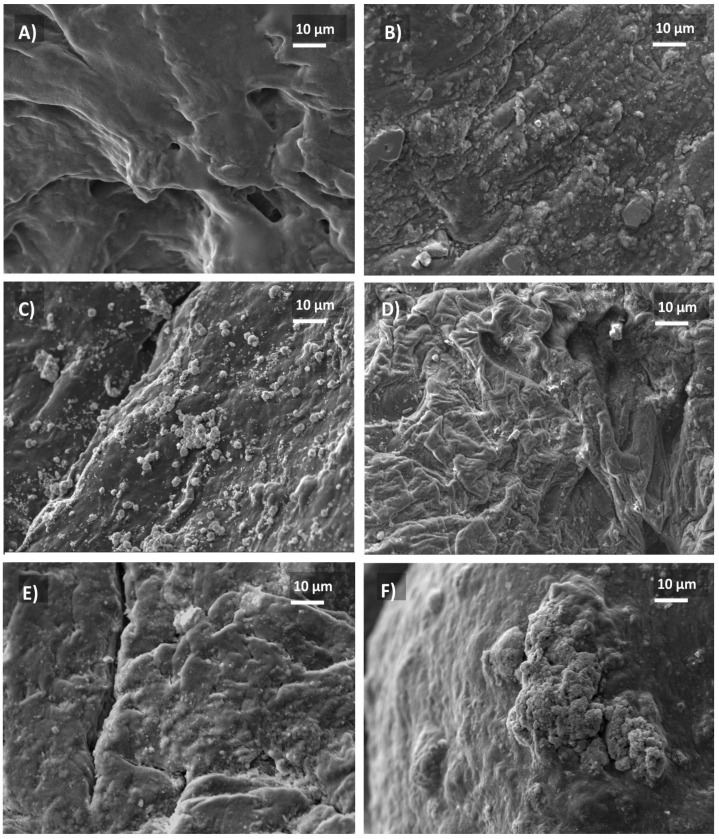
Micrographs of samples after soaking 21d in body fluids simulated (**A**) AG1 (control); (**B**) AG2 (Al_2_O_3_); (**C**) AG3 (Na_2_Ti_6_O_13_); (**D**) AG4 (TiO_2_); (**E**) AG5 (ZrO_2_); and (**F**) AG6 (WO_3_).

SEM studies. All samples were crosslinked in calcium chloride soliton and soaked for 21 days in simulated physiological fluids as in the methodology, as referenced. In Figure 3A, hydrogel AG1 shows a discrete hole (~15 μm) and a smooth surface. Figure 3B photomicrography shows a rough surface, particles of 5–10 μm over the surface. In Figure 3C, white round particles are deposited with a size of 2–3 μm. In Figure 3D, a semi-rough surface is observed; Figure 3E shows that few particles on an uneven surface are observed. However, in Figure 3F, a rough layer is deposited over the substrate and a layer with high calcium and phosphate concentration content is exhibited, extracted by EDS. The analysis of energy-discursiveness of AG2, AG3, AG4, AG5, and AG6 substrates shows contents of elemental calcium and phosphorus; the Ca/P are higher than those expected for hydroxyapatite (1.67). This study can also be observed in Table 2. In addition, sodium chloride content is still observed from physiologic fluids. For comparative evaluation of substrates, AG3 and AG6 presents larger particles and a cover, respectively, in the surface.

Dynamic light scattering. An analysis of size particles was observed, and data are collected in Table 2. Suppliers’ sizes are smaller than those measured; this effect could be due to an electrostatic agglomeration; in contrast, particles synthesized are the smallest. AG3 was synthesized by the sol-gel method and calcined at 1250 °C, and values are approximately 200.5 nm in size. In AG6, the powder was obtained by the precipitation technique and calcined at 500 °C, and the size is approximately 210.2 nm. A former study from Urruelas et al. analyzed the effect of different filler concentrations (0.0, 0.05, and 0.1). A non-effect in the viscosity of the solution was observed. Furthermore, good viscoelastic modulus performance was observed with the addition of 0.02 *w/v* CaCl_2_ for extrusion purposes [19]. For instance, higher concentrations of crosslinking agents resulted in higher viscoelastic modulus, as is desired [43].

**Figure 4:**
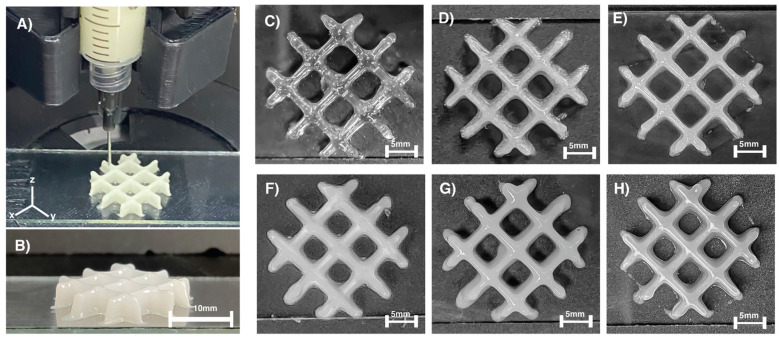
Extrusion of alginate-gelatin samples (**A**) Isometric view of the 3D printed structure; (**B**) lateral view; (**C**) AG1 (control); (**D**) AG2 (Al_2_O_3_); (**E**) AG3 (Na_2_Ti_6_O_13_); (**F**) AG4 (TiO_2_); (**G**) AG5 (ZrO_2_); and (**H**) AG6 (WO_3_).

Extrusion-based printing. For extrusion purposes, a 12% CaCl_2_ pre-crosslinked solution was used. Figure 4 shows six different samples with the same concentrations of particles that were successfully fabricated with a custom-designed 3D printer. 3D printing of all the hydrogel structures used a flow rate of 40% over a 76 × 25 × 1.02 mm coverslip. In addition, CaCl_2_ was added in small amounts before extrusion to activate the hydrogel’s cross-linking partially. As mentioned earlier, the viscosity plays an essential role in the stability of the hydrogels during the extrusion process, since the more layers that overlap, the greater the pressure exerted on the hydrogel, so, in some cases, part of the first layer printed could begin to expand slightly on the coverslip as can be seen in practically all the sections of Figure 4. Regardless, none of the structures entirely collapsed during the extrusion process. Further increasing the percentage of cross-linking of the hydrogels before its extrusion to increase the hydrogel’s stability during extrusion turns out to be practically impossible because this would cause clogging in the extruder needle. In addition, it is important to mention that 3D printing is crucial to develop a layer-by-layer system that can be used as a method of biofabrication, as cells could potentially could be extruded for 2D and 3D culture systems, in comparison with less robust methodologies as molding that lacks the complexity for crucial biomedical applications.

**Figure 5:**
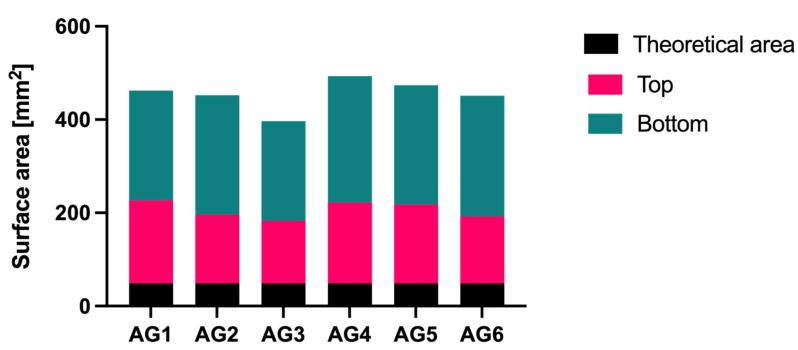
The surface area of the alginate-gelatin samples, AG1 (control), AG2 (Al_2_O_3_), AG3 (Na_2_Ti_6_O_13_), AG4 (TiO_2_), AG5 (ZrO_2_), and AG6 (WO_3_).

Due to the deposition onto the substrate, a decrease in the shear rate caused a sharp increase in viscosity resulting in thinning materials and shape fidelity [44], as is shown in Figure 4C,E,H). In addition, some structures collapsed during the additive process due to the low viscosity of the hydrogel solution (Figure 4F,G). Measurements of the top and bottom layers of the hydrogels were taken and compared to the theoretical area from the 3D model (cross-section area in mm^2^) to understand the impact of the settlement of the printed hydrogels (see Figure 5). Considering these measurements, AG3 presented the most accurate structure due to the smallest particle size of the *Na_2_Ti_6_O_1_*_3_, where surface areas on the top (135.06 mm^2^) and the bottom (214.28 mm^2^) were the closest to the theoretical surface area. On the other hand, AG6 presented the highest surface area difference between the top (173.72 mm^2^) and the bottom (270.97 mm^2^) due to the weight of the previous layers and the low viscosity of the hydrogel solution [44,45]. Consistency obtained in all samples was achieved by using optimal processing parameters and a pre-cross-linking solution where a mean of 36% and standard deviation of 6.57% in the difference of top and bottom surface areas were obtained.

**Figure 6:**
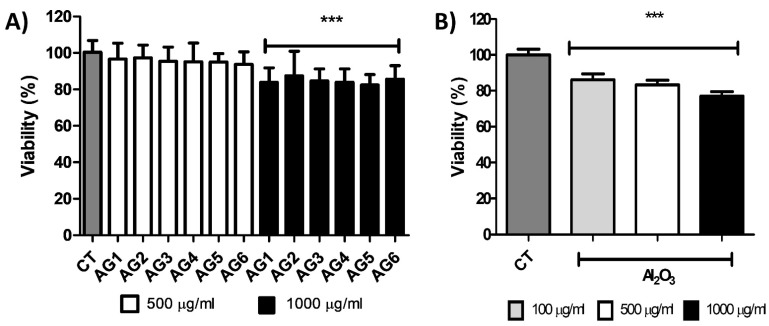
Cell viability after 24-h exposure of composites (**A**) AG1 (control), AG2 (Al_2_O_3_), AG3 (Na_2_Ti_6_O_13_), AG4 (TiO_2_), AG5 (ZrO_2_), and AG6 (WO_3_) or (**B**): free Al_2_O_3_. Values are means ± SD, *n* = 3; *** *p* < 0.0001 vs. CT. CT = Cells without any composite or free Al_2_O_3_.

Cell viability. To assess the effect of the selected materials composite of Alg/Gel and bioceramic on endothelial cells viability, we cultured C166 cells with different concentrations of composites (0–500 and 1000 µg/mL). We examined cell death after 24 h treatment using Alamar blue. As shown in Figure 6A, only concentrations as high as 1000 µg/mL induced significant cytotoxic effects in vitro, with an approximately 10% viability reduction, indicating tolerance and reduced toxicity of composites below that concentration (Figure 6). The use of Al_2_O_3_, Na_2_Ti_6_O_13_, TiO_2_, ZrO_2_, and WO_3_ particles embedded in an alginate-gelatin matrix showed that these composites could be initially used as a scaffold to promote the hydroxyapatite nucleation, without the side effects of the free ceramics, which has been well characterized for its toxicity properties in different cells and tissues, even at low concentrations of nano and microparticles of TiO_2_ or SiO_2_, or free Al_2_O_3_ as we tested (Figure 6B). To assess the effect of a free bioceramic on cell viability, we cultured C166 cells with different concentrations of Al_2_O_3_ (100, 500, and 1000 µg/mL). We examined cell death after 24 h of treatment using Alamar blue, and a cytotoxic effect was observed at initial concentrations of 100 µg/mL, indicating a reduced metabolic activity at those conditions. As different groups have shown, free ceramics exerts higher side effects, such as nano or microparticles, e.g., SiO_2_ [46,47] in different cells and tissues, even at low concentrations, as we tested with free Al_2_O_3_ (Figure 6B).

## 4. Conclusions

In summary, we explored a novel synthesis by the sol-gel technique for *Na_2_Ti_6_O_13_* and *WO_3_* studied in our research group and pre-synthesized bioceramics such as *Al*_2_*O*_3_, *TiO*_2_, and *ZrO*_2_. These bioactive particles were integrated into a biopolymer base of Alginate/Gelatin as a scaffold. This study relied on characterization techniques XRD, FT-IR, TGA, DLS, SEM, mechanical properties, and biological assessments. Moreover, we compare the reinforcement between all composites of polymer-bioceramic without particles. Therefore, one of the first findings was that the higher the molecular weight of the system, the stronger mechanical strength was observed, as seen with our synthesized sol-gel particles *Na_2_Ti_6_O_1_*_3_, and *WO_3_.* At the same time, these were the smallest particles during the DLS evaluation, causing a more efficient dispersion of the particles, as they had lower particle diameter than the pre-synthetized particles. XRD studies and FTIR analysis confirmed the chemical nature of the alginate-gelatin matrix reinforced with different particles; principal intensities are observed in Figure 1. The infrared analysis confirmed the presence of typical bands in those biopolymers with phosphate carbonated bands. Mechanical properties were performed; all reinforced samples showed an enhanced strength compared to control AG1, 20–60 kPa. Although all the constructs were 3D printable, *Na_2_Ti_6_O_13_* presented the best accuracy to the shape. Consequently, once the samples were soaked in simulated body fluids for 21 days, the systems presented calcium, phosphate, chloride, and sodium content, corroborated with EDAX analysis. Finally, this hydrogel system generates a polymeric-bioceramic scaffold that could be implanted in damaged tissues for bone defects resulting from chronic infections such as periodontal diseases and congenital disabilities. For future works, further characterizations are needed to understand its biological behavior and printability under more complex conditions will be performed. Therefore, with all these efforts, we could be one step closer to one of the current alternatives that can solve the current issues needed in the biomedical engineering field.

## Figures and Tables

**Table 1:** Label samples by their composition.

%Alginate	Hydrogels % Gelatin	1% Bioceramic Filler	Label
3.5	0.88	-	AG1
3.5	0.88	Al_2_O_3_ *	AG2
3.5	0.88	Na_2_Ti_6_O_13_	AG3
3.5	0.88	TiO_2_ *	AG4
3.5	0.88	ZrO_2_ *	AG5
3.5	0.88	WO_3_	AG6

* commercial.

**Table 2:** Size from bioceramics used for this comparative study and EDAX data.

Sample	AG1	AG2	AG3	AG4	AG5	AG6
Bioceramic Filler		*Al_2_O_3_*	*Na_2_Ti_6_O_13_*	*TiO_2_*	*ZrO_2_*	*WO_3_*
Particle Size (nm)	-	516.2	200.5	652.2	459.2	210.2
Oxygen (%)		57.13	77.64	65.65	71.28	77.83
Calcium (%)		14.4	9.38	-	11.66	10.56
Sodium (%)		12.63	6.04	8.88	6.89	6.14
Chlorine (%)		14.83	4.18	-	4.61	2.88
Phosphorus (%)		2.80	0.99	-	3.38	2.13
Potassium (%)		0.46	-	-	1.4	-
Titanium (%)		-	0.43	23.69	-	-
Aluminum (%)		-	0.57	1.78	-	-
Silicon (%)		-	0.34	-	-	-
Ca/P (%)	-	5.14	9.47	-	3.45	4.9

## Supplementary Materials

The following are available online at https://www.mdpi.com/article/10.3390/gels7040184/s1, Information related to the characterization of *Na2Ti6O_13_* and *WO_3_* is presented in the supplementary document.
