# Supplementary material for: Reinforcement of Alginate-Gelatin Hydrogels with Bioceramics for Biomedical Applications: A Comparative Study
Source: Gels. 2021 Oct 26;7(4):184. doi: 10.3390/gels7040184 (PMC8628790; doi:10.3390/gels7040184)
Supplement: Supplementary file 1 [file gels-07-00184-s001.zip › gels-1434836-supplementary.pdf]

Article.

# Reinforcement of Alginate-Gelatin hydrogels with Bioceramics for biomedical applications: a comparative study

**Alan Avila-Ramirez[1, 2], Kevin Katzim-Rios[1] , Carlos Enrique Guerrero-Beltrán [3], Erick Ramírez-Cedillo[1] , Wendy Ortega-Lara [1,*]**

[1] Tecnologico de Monterrey; Escuela de Ingeniería y Ciencias
[2] Division of Biological & Environmental Science & Engineering (BESE), King Abdullah University of Science and Technology (KAUST), Thuwal 23955-6900, Saudi Arabia
[3] Tecnologico de Monterrey, Escuela de Medicina y Ciencias de la Salud, Medicina Cardiovascular y Metabolómica, Monterrey, N.L. México; enriqueguerrero@tec.mx
* Correspondence: wlortega@tec.mx;

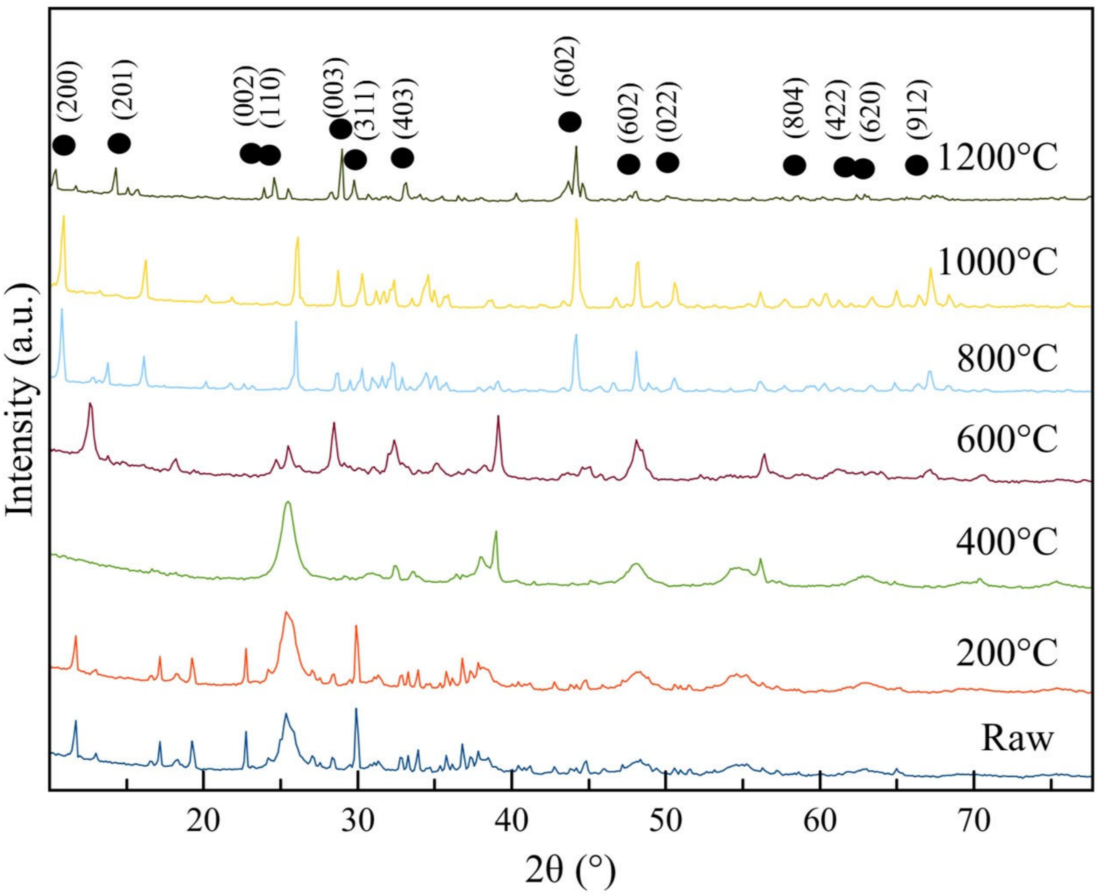


**$Na_2Ti_6O_{13}$**

**Figure S1. X-ray diffractogram of $Na_2Ti_6O_{13}$ synthesis with different temperatures of calcination until 1200°C**

The pronounced peaks of figure S1 indicate the XRD patterns of the samples synthesized from sodium acetate and titanium butoxide (IV), which are indexed to $Na_2Ti_6O_{13}$ phase (monoclinic structure) space group. Prominent peaks belong to Anatase ($TiO_2$) in lower temperatures, indicating the starting materials have been almost turning into $Na_2Ti_6O_{13}$ products at 1200°C. At lower temperatures below 1000°C it can be seen different peaks corresponding to Anatase ($TiO_2$), which peaks crystal structure (2theta) at 25 °, 39°and 47°, obtained from ISCD (01-071-1168), and Rutile ($TiO_2$) since 600 °with 27°, 48°and 56°, before the desired crystal at the 1200°C of calcination(*1*) (*2*).

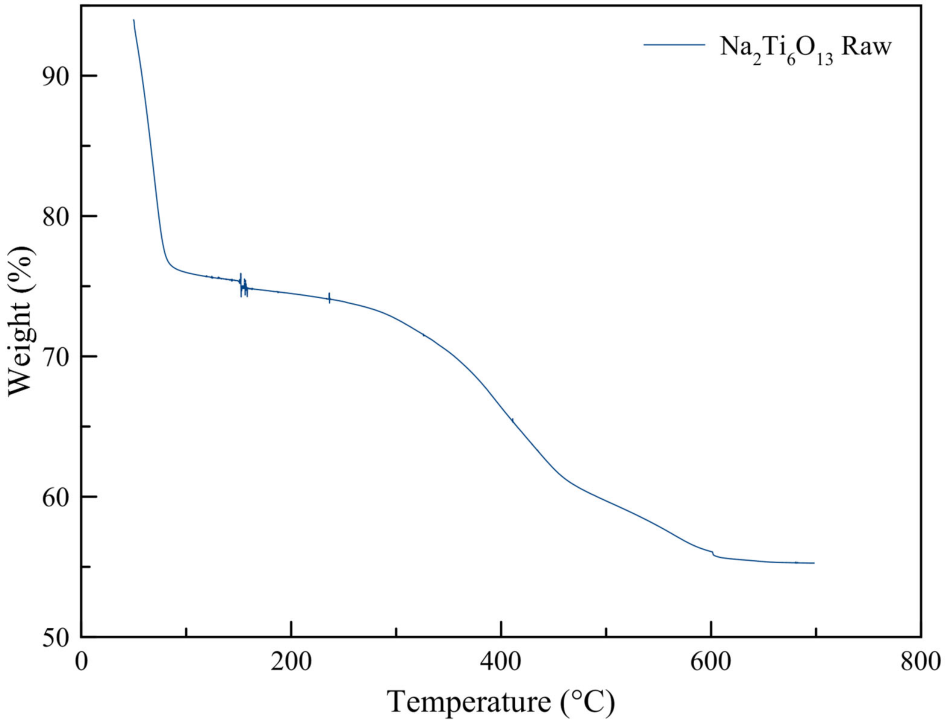


**Figure S2. Thermogravimetric analysis spectra from pre-calcined $Na_2Ti_6O_{13}$**

The evolution of gravimetric loss is elucidated in the range from around 90°C to 150°C the last of 25% of weight due to the loss of water and hydroxyl groups that are coordinated to the initial pre-calcined samples due to solvents as tert-butanol, deionized water, and coordinated water bonds from sodium acetate that are used in thermal synthesis step before calcination at high temperatures. A second weight-loss of 30% from 250°C to 600°C represents the loss of carbonyl from sodium acetate and by the rearrangements and loss of oxygen atoms until getting the desired structure (*3*).

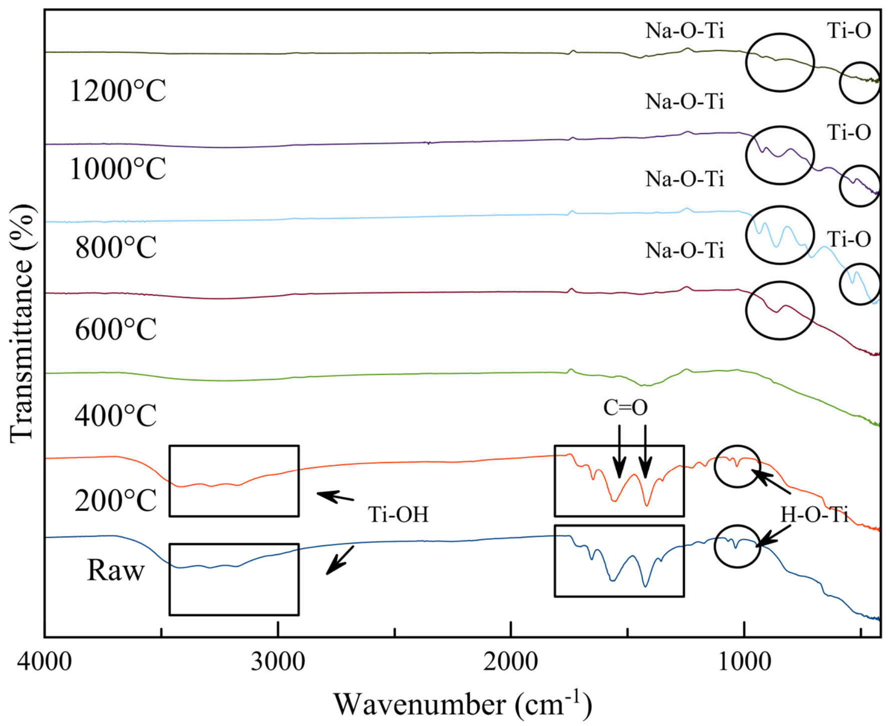


**Figure S3. FT-IR spectra from pre-calcined raw $Na_2Ti_6O_{13}$ material to calcined higher temperatures**

FT-IR studies from figure S5 show that the bands at 1730 $cm^{-1}$ and 3150 $cm^{-1}$ are related to the bending and stretching modes of the chemisorbed or interlamellar water molecules. It is essential to mention that the shoulder at 2900 $cm^{-1}$ belongs to the interaction between Ti-OH ions at temperatures below 400 °C. When the temperature increases in post-synthesis, that the signal from water was removed, and the Ti–OH bonds were modified Sodium titanates contain Ti–O and Ti-OH is stretching and bending vibration bands with different intensities in the region 500–1000 $cm^{-1}$. The peak at 910 $cm^{-1}$ corresponds to Na-O-Ti, and bonds in $TiO_6$ octahedra appear at 600 °C and above this temperature. The small peak at 974 $cm^{-1}$ is due to the bending vibrations of Ti–OH non-bridging bonds in the as-made sample. The alkali–oxygen vibrations usually provide absorption bands at 1440, 1370, 1060, and 880 $cm^{-1}$. The short peaks near 500 $cm^{-1}$ correspond to Ti-O when achieving calcination up to 600°C or higher temperatures until 1200°C. The sharp peak at 1460 $cm^{-1}$ of the dehydrated sample is attributable to new alkali–oxygen (Na–O). The low-intensity broadband in the 3200–3600 $cm^{-1}$ region and the band centered at 1625 $cm^{-1}$ are assigned to O–H stretching and deformation vibrations of weakly bound water, giving evidence for the existence of Ti–OH or Ti=O groups due to Titanium(IV) butoxide, that was used in the synthesis protocol as the precursor of titanium ions meanwhile the two bands with high intensity at raw, and 200 °C correspond to carbonyl groups which are from the sodium acetate that disappear at higher temperatures. Peaks corresponding to the bending vibrations of the Ti–O groups at 506 and 448 $cm^{-1}$ converge into a broader peak centered at around 480 $cm^{-1}$ seen from 800 °C to 1200 °C; this by the elimination of hydration and the rearrangement of atoms. The shoulder at 878 $cm^{-1}$ is characteristic of the Na–O bending of the Na–O–Ti bond of sodium titanates, whereas the minimal signal at 1061 $cm^{-1}$ can be attributed to the respective bending vibration H–O–Ti of the hydroxyl titanates (*4*, *5*), (*6*), (*7*).

**$WO_3$**

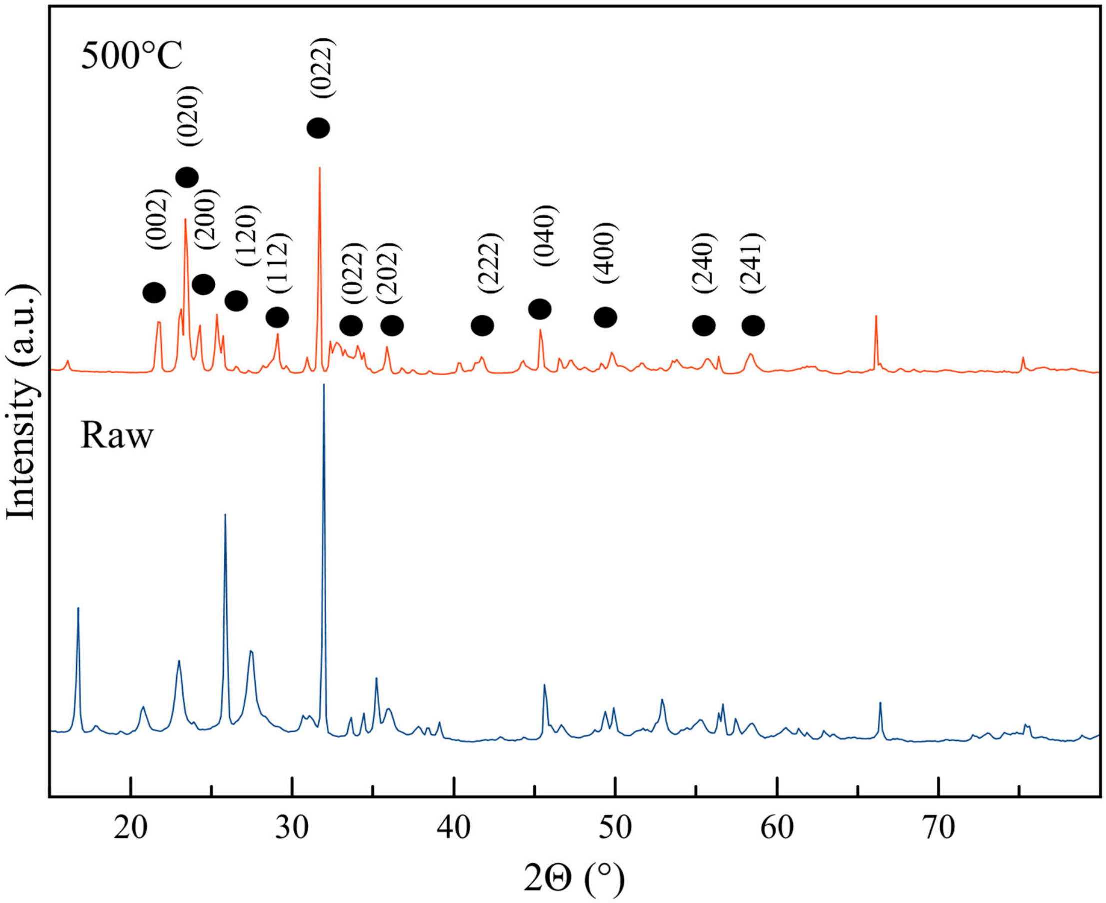


**Figure S4. X-ray diffraction from the synthesis of raw pre-calcined and 500 °C calcination of $WO_3$**

From the XRD pattern of Fig S2, $WO_3$ particles calcined at 500°C are monoclinic crystals has their highest (022) peak was at 34°with low intensity of (020) at peak 23°peak, while a peak with increased intensity of $WO_3$ (200) at 24°. The previous information is obtained from ICSD (00-024-0747). $WO_3$ showed balanced peak intensity among all three samples, indicating the crystal's presence (*8*), (*9*). In the case of the structure of the raw pre-calcinate material showing residues of $NaWO_3$ dehydrated, that is presented in the 2tetha 16°and 27°(*10*).

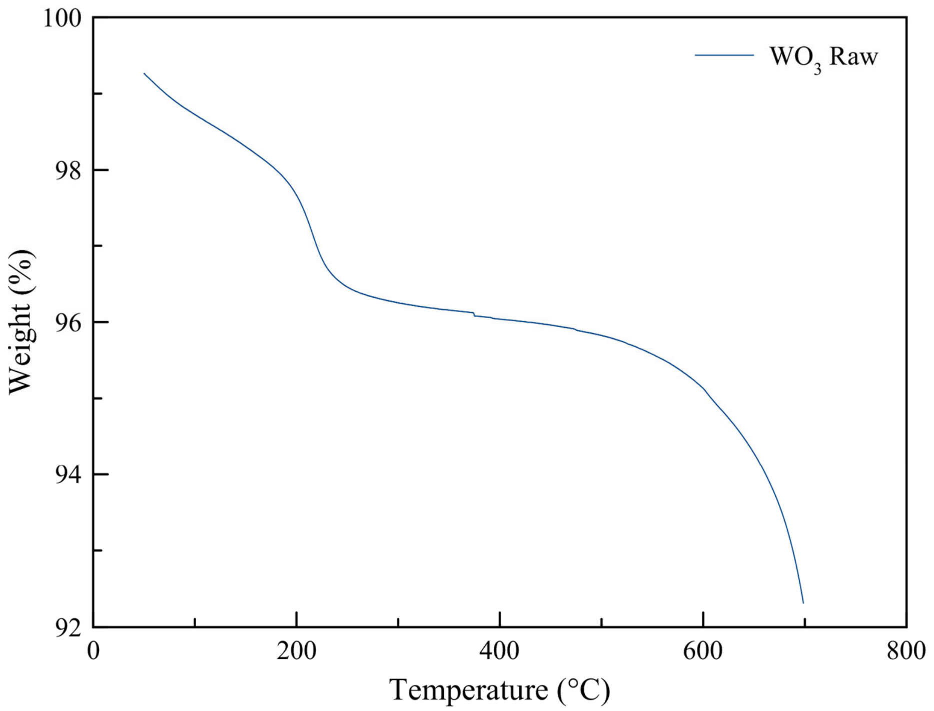


**Figure S5. Thermogravimetric analysis spectra from pre-calcined $WO_3$**

In the thermogravimetric analysis from figure S4 of $WO_3$, before 200°C the decay of the slope corresponds to the elimination of the coordinated water molecules attached initially by the sodium tungstate dihydrate precursor. There is a constant behavior until the degradation at 600°C. Therefore, while synthesizing the particles at 500°C, the final structure does not lose a significant percentage of weight from 200°C to 600°C (*11*).

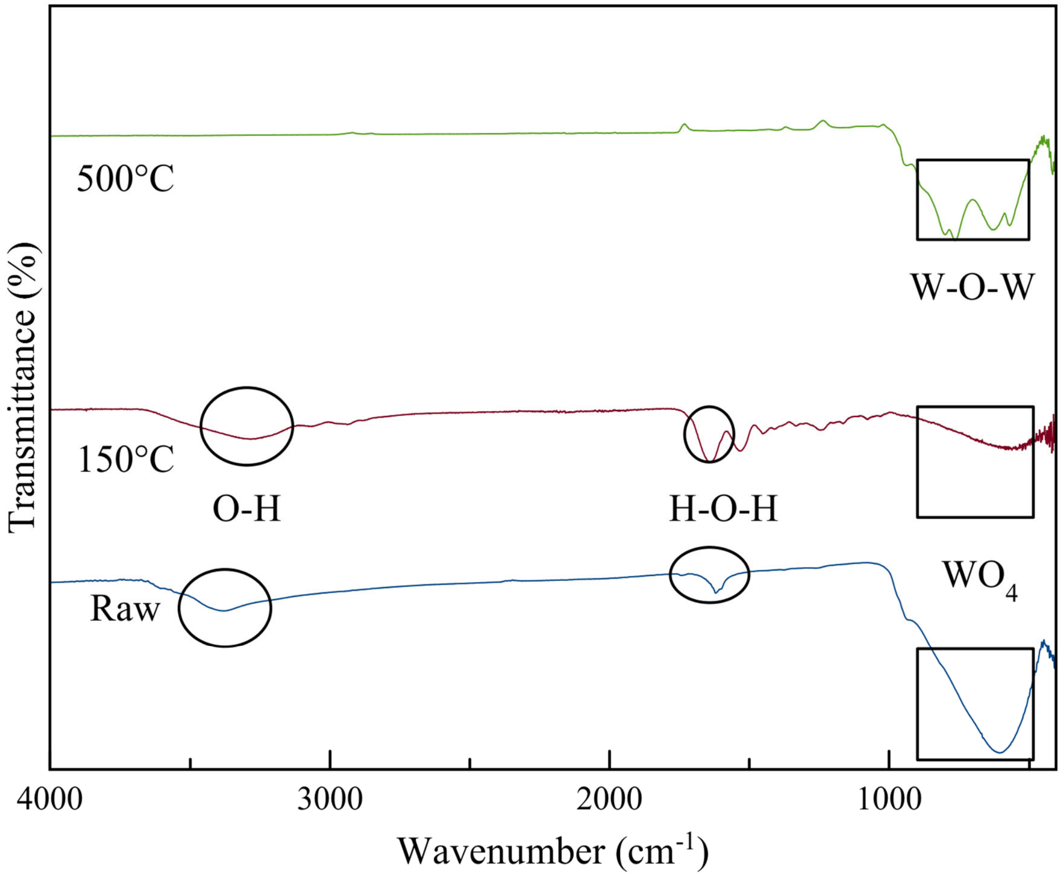


**Figure S6. FT-IR from $WO_3$ pre-calcined and with different temperatures of calcination**

The higher bands at the 3700–3100 $cm^{-1}$ region are observed due to OH groups and adsorbed water. Specifically, the broad bands at 3450 and 3692 $cm^{-1}$ are due to various O–H stretching modes and a sharp mode at 1620 $cm^{-1}$ is the H-O-H bending mode. Conversely, no bands were observed in the region 3400–3700 $cm^{-1}$ or at 1620 $cm^{-1}$ when calcined at higher temperatures. The shallow or superficial shoulder, located around 600 $cm^{-1}$ in the pre-calcined powders, can refer to the initial $WO_4$ from the tungstate precursor. The W-O peak shifts towards higher wavenumbers, at 813 $cm^{-1}$, where this shift corresponds to the formation of W-O-W crystalline bonds that gives information about the changes, from raw to calcined powders 500°C (*12*) (*13*).

## References


1. Luo, L., Zhen, Y., Lu, Y., Zhou, K., Huang, J., Huang, Z., Mathur, S., and Hong, Z. (2020) Structural evolution from layered Na2Ti3O7 to Na2Ti6O13 nanowires enabling a highly reversible anode for Mg-ion batteries, *Nanoscale*.
2. (2018) Titanium butoxide molar ratio effect in the TiO2 nanoparticles size and methylene blue degradation, *Optik* , Urban & Fischer *157*, 890–894.
3. Torres-Martínez, L. M., Juárez-Ramírez, I., Del Ángel-Sánchez, K., Garza-Tovar, L., Cruz-López, A., and Del Ángel, G. (2008) Rietveld refinement of sol–gel Na2Ti6O13 and its photocatalytic performance on the degradation of methylene blue, *Journal of Sol-Gel Science and Technology*.
4. Shirpour, M., Cabana, J., and Doeff, M. (2013) New materials based on a layered sodium titanate for dual electrochemical Na and Li intercalation systems, *Energy Environ. Sci.*, The Royal Society of Chemistry *6*, 2538–2547.
5. Jian, Z., Huang, S., and Zhang, Y. (2013) Photocatalytic Degradation of 2,4-Dichlorophenol Using Nanosized Na2Ti6O13/TiO2 Heterostructure Particles, *Int. J. Photoenergy*, Hindawi *2013*.

6. (2004) Synthesis and characterization of sodium titanates Na2Ti3O7 and Na2Ti6O13, *J. Solid State Chem.*, Academic Press *177*, 4508–4515.
7. Tsiourvas, D., Tsetsekou, A., Arkas, M., Diplas, S., and Mastrogianni, E. (2010) Covalent attachment of a bioactive hyperbranched polymeric layer to titanium surface for the biomimetic growth of calcium phosphates, *J. Mater. Sci. Mater. Med.*, Springer *22*, 85–96.
8. Vuong, N. M., Kim, D., and Kim, H. (2015) Porous Au-embedded WO3 Nanowire Structure for Efficient Detection of CH4 and H2S, *Sci. Rep. 5*, 11040.
9. Adhikari, S., and Sarkar, D. (2013) Electrochemical Response for Spherical and Rod Shaped WO3 Nanoparticles, *ISRN Nanotechnology*.
10. (2015) Characterization of Mn–Na2WO4/SiO2 and Mn–Na2WO4/MgO catalysts for the oxidative coupling of methane, *Appl. Catal. A*, Elsevier *497*, 96–106.
11. Huang, J., Xu, X., Gu, C., Yang, M., Yang, M., and Liu, J. (2011) Large-scale synthesis of hydrated tungsten oxide 3D architectures by a simple chemical solution route and their gas-sensing properties, *J. Mater. Chem.*, The Royal Society of Chemistry *21*, 13283–13289.
12. (2015) Electrochemically synthesized tungsten trioxide nanostructures for photoelectrochemical water splitting: Influence of heat treatment on physicochemical properties, photocurrent densities and electron shuttling, *Colloids Surf. A Physicochem. Eng. Asp.*, Elsevier *484*, 297–303.
13. (2015) NO2 Gas Response of WO3 Nanofibers by Light and Thermal Activation, *Procedia Engineering*, Elsevier *120*, 791–794.
